# The *CNDP1* (CTG)_5_ Polymorphism Is Associated with Biopsy-Proven Diabetic Nephropathy, Time on Hemodialysis, and Diabetes Duration

**DOI:** 10.1155/2017/9506730

**Published:** 2017-05-03

**Authors:** Thomas Albrecht, Shiqi Zhang, Jana D. Braun, Li Xia, Angelica Rodriquez, Jiedong Qiu, Verena Peters, Claus P. Schmitt, Jacob van den Born, Stephan J. L. Bakker, Alexander Lammert, Hannes Köppel, Peter Schnuelle, Bernhard K. Krämer, Benito A. Yard, Sibylle J. Hauske

**Affiliations:** ^1^Fifth Medical Department (Nephrology/Endocrinology/Rheumatology), University Medical Center Mannheim, University of Heidelberg, Mannheim, Germany; ^2^Department of Endocrinology, The First Affiliated Hospital of Anhui Medical University, Hefei, China; ^3^Centre for Pediatric and Adolescent Medicine, University of Heidelberg, Heidelberg, Germany; ^4^Nephrology, University Medical Center Groningen, University of Groningen, Groningen, Netherlands

## Abstract

Considering that the homozygous *CNDP1* (CTG)_5_ genotype affords protection against diabetic nephropathy (DN) in female patients with type 2 diabetes, this study assessed if this association remains gender-specific when applying clinical inclusion criteria (CIC-DN) or biopsy proof (BP-DN). Additionally, it assessed if the prevalence of the protective genotype changes with diabetes duration and time on hemodialysis and if this occurs in association with serum carnosinase (CN-1) activity. Whereas the distribution of the (CTG)_5_ homozygous genotype in the no-DN and CIC-DN patients was comparable, a lower frequency was found in the BP-DN patients, particularly in females. We observed a significant trend towards high frequencies of the (CTG)_5_ homozygous genotype with increased time on dialysis. This was also observed for diabetes duration but only reached significance when both (CTG)_5_ homo- and heterozygous patients were included. CN-1 activity negatively correlated with time on hemodialysis and was lower in (CTG)_5_ homozygous patients. The latter remained significant in female subjects after gender stratification. We confirm the association between the *CNDP1* genotype and DN to be likely gender-specific. Although our data also suggest that (CTG)_5_ homozygous patients may have a survival advantage on dialysis and in diabetes, this hypothesis needs to be confirmed in a prospective cohort study.

## 1. Introduction

Diabetic nephropathy (DN) occurs in approximately 40% of patients with type 1 and type 2 diabetes [1] and is the leading cause of end-stage renal disease (ESRD) [2]. Compelling evidence has shown that susceptibility to DN is genetically determined [3, 4]. Amongst the reported linkage studies, there seems to be consistency in the linkage between human chromosome 18q22.3-q23 and DN [4–7]; linkage to the DN trait on chromosomes 7q21.3, 10p15.3, and 14q23.1 has also been reported [7]. Linkage with 18q22.3 was observed in populations of different ethnicities, for example, American Indians [8], Afro-Americans [9], and Caucasians [5].

Janssen et al. initially postulated that the *CNDP1* gene on chromosome 18q22.3-q23, encoding serum carnosinase (CN-1), is a susceptibility gene for DN in type 2 diabetes mellitus (T2DM) patients [10]. It was found that T2DM patients homozygous for the *CNDP1* (CTG)_5_ allele are less frequently affected by DN compared to T2DM patients with other *CNDP1* genotypes [10]. The prevalence of the (CTG)_5_ allele strongly varies with different ethnicities. While homozygosity for the (CTG)_5_ allele is more frequent in the European population (38.6% in healthy controls and 29.3% in diabetic patients with ESRD) [11], this genotype seems to be much more rare in the Asian population with a high prevalence of DN [12, 13]. It has also been reported that the association between the *CNDP1* genotype and DN is sex-specific and independent of susceptibility to T2DM [14].

Since most patients with T2DM are not formally evaluated with a renal biopsy, the diagnosis of DN is based on clinical criteria, for example, persistent macroalbuminuria on at least 2 independent occasions (albumin excretion rate > 300 mg/d or >200 mg/l or ACR (albumin/creatinine ratio) > 300 mg/g). Yet, biopsy-based retrospective evaluations of the prevalence of nondiabetic renal disease (NDRD) in T2DM patients revealed a high percentage of patients having NDRD without evidence of concurrent DN [15, 16]. Although the predictive value of clinical criteria for DN in T2DM patients can be improved by the presence of proliferative retinopathy [17, 18], genetic studies that use clinical inclusion criteria (CIC) for group allocation still bear the risk of wrongly assigning patients to the DN group. In the present study, we assessed if the association between *CNDP1* and DN is still observed when applying CIC or biopsy-proven diabetic nephropathy (BP-DN) and, if so, whether this is only observed in female T2DM patients. Since also an association between the *CNDP1* (CTG)_5_ homozygous genotype and cardiovascular mortality has been reported to be sex-specific [19], we also assessed if the prevalence of this genotype changes with diabetes duration and time on dialysis. Since T2DM patients on dialysis have a high mortality risk, it would be expected that the proportion of *CNDP1* (CTG)_5_ homozygous patients would decrease with time on dialysis, particularly in females.

## 2. Materials and Methods

### 2.1. Patients

**Figure 1:**
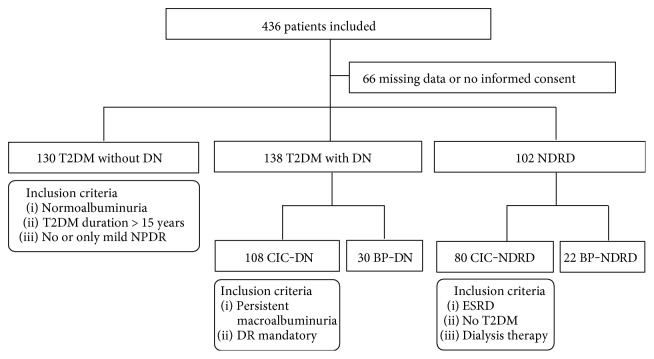
Flow diagram for patient recruitment and group allocation. DN: diabetic nephropathy, NDRD: nondiabetic renal disease, NPDR: nonproliferative diabetic retinopathy, CIC: clinical inclusion criteria, BP: biopsy-proven, DR: diabetic retinopathy, ESRD: end-stage renal disease.

Patients were recruited between 2011 and 2014 from the Fifth Medical Clinic and Dialysis Unit at the University Medical Centre Mannheim and different nephrology practices in proximity (Centre for Renal Disease, Weinheim, Lindenfels, Viernheim; Nephrocare Ludwigshafen GmbH; KfH Nierenzentrum, Ludwigshafen; Nephrology Practice Frankenthal, Bad Dürkheim, Lampertheim). After screening of clinical records (*n* = 384) and patients with a biopsy-proven renal diagnosis (*n* = 52), a total of 436 patients were deemed to be eligible for this study. Due to the retrospective nature of this study, indications for renal biopsies were not uniform and the histological evaluation was undertaken by different pathologists. Out of the 436 initially selected patients, 66 were excluded because of incomplete clinical data or missing informed consent. The remaining patients (*n* = 370) were allocated to 5 different groups, that is, 130 T2DM patients without DN (no-DN), 108 T2DM patients with CIC-DN, 30 T2DM patients with BP-DN, 80 patients with CIC nondiabetic renal disease (CIC-NDRD), and 22 patients with biopsy-proven nondiabetic renal disease (BP-NDRD) (Figure 1()). Patients without renal biopsy material were allocated on the basis of clinical criteria as specified below, whereas patients with renal biopsy material were allocated either to the BP-DN or to the BP-NDRD group. The ethnicity distributions in the no-DN and BP-DN groups were broadly comparable (no-DN: 75% from Germany, 12.5% from Turkey; BP-DN: 70% from Germany, 17% from Turkey).

Inclusion criteria for clinical diagnosis of DN were as follows: persistent macroalbuminuria on at least 2 independent occasions (albumin excretion rate > 300 mg/d or >200 mg/l or ACR (albumin/creatinine ratio) > 300 mg/g) in combination with the diagnosis of diabetic retinopathy (DR) (all severity degrees were allowed). This combination was obligatory to reduce the possibility that cases with proteinuria due to renal disease other than DN (NDRD) were included [20]. Anuric patients with a history of macroalbuminuria were included. Exclusion criteria were urinary tract infection or fever at the time of urine investigation, documented renal disease other than DN, and a history of kidney transplantation.

T2DM patients without DN (=control group) fulfilled the following criteria: diabetes duration of at least 15 years accompanied by normoalbuminuria on at least two independent occasions (albumin excretion rate < 30 mg/d or <20 mg/l or ACR< 30 mg/g). Since approximately 80% of the diabetic patients in this group were on ACE inhibitors or AT_1_ blockers, false-negative results based on albuminuria could not be excluded. However, to minimize the possibility of DN patients in the control group, only the normoalbuminuric patients with no or mild nonproliferate DR were included since the presence of nephropathy without significant DR is rare [18, 21, 22]. Diabetes mellitus was defined by a documented history of diabetes or a fasting blood glucose of ≥7.0 mmol/l (126 mg/dl), a casual plasma glucose level of ≥11.1 mmol/l (200 mg/dl), or a HbA1c level of ≥6.5%.

Nondiabetic patients with ESRD were included in the CIC-NDRD group (*n* = 80) after screening of patient records and medications along with laboratory testing for plasma glucose or HbA1c levels to exclude diabetes.

Out of the whole cohort, 175 patients were on hemodialysis including 90 diabetic patients and 85 NDRD patients. Patients were dialyzed using a blood flow rate of 200–300 ml/min and a dialysate flow rate of approximately 500 ml/min.

Estimated GFR was calculated based on the MDRD formula [23]. Serum was used to measure CN-1 activity and concentration. Genotyping was performed on EDTA blood. All samples were stored at −20°C until use. The study protocol was approved by the local ethics committee, and all patients gave written informed consent prior to the study enrollment (no. 0193/2001).

### 2.2. Genotyping

Genomic DNA was isolated from whole blood using the Genomic DNA isolation kit (Promega, Mannheim, Germany) according to the manufacturer's instruction and stored at −20°C until use. A 167-base pair fragment spanning the (CTG)_*n*_ polymorphism of *CNDP1* was amplified by standard PCR methods using a fluorescence labeled forward primer (5′FAM-AGGCAGCTGTGTGAGGTAAC-3′) and an unlabeled reverse primer (5′-GGGTGAGGAGAACATGCC-3′), respectively. Genotyping was performed by means of fragment analysis on an ABI 310 sequencing platform (ABI PRISM DNA analyzer 3100).

### 2.3. CN-1 Activity Assay

CN-1 activity was assayed based on the method described by Teufel et al. [24].

### 2.4. Statistical Analysis

Quantitative data are depicted as median with corresponding 25th and 75th percentiles (interquartile range) or, when appropriate, as mean ± SEM. Student's *t*-test was carried out for comparison of continuous variables. Qualitative data were analyzed using the *χ*^2^ test. For pairwise comparisons, frequency tables were partitioned into respective 2 × 2 subtables. The significance level was corrected using the Bonferroni method based on the number of planned comparisons. Univariate and multivariate logistic regression analyses were performed to examine predictors of biopsy-proven diabetic nephropathy. Variables with a *P* value of <0.25 in the univariate analysis were included into a full-model multivariate analysis. To compare frequencies among groups, which have an ordering, the *χ*^2^ test for trend (Cochran-Armitage test for trend) was carried out. Time on hemodialysis was logarithmically transformed before the correlation analysis because of its skewed distribution. The significance level *α* was defined as 0.05. Statistical analyses were performed with GraphPad Prism 6.0 (GraphPad Software, Inc., La Jolla, California) and Microsoft Excel/XLSTAT 19.01 (Addinsoft, New York, USA).

## 3. Results

### 3.1. Patient Characteristics

Demographic and clinical characteristics of all studied individuals are presented in Table 1. Significantly more male patients (64%) were recruited in the CIC-DN group (males: *n* = 69, females: *n* = 39) as compared to the no-DN group (50%) (males: *n* = 65, females: *n* = 65). Gender distribution in the other groups resembled the CIC-DN group (CIC-NDRD: 61% males, BP-DN: 70% males and BP-NDRD: 64% males). BP-NDRD patients were diagnosed with hypertensive nephrosclerosis, IgA nephropathy, lupus nephritis, or other categories of glomerulonephritis (e.g., granulomatosis with polyangiitis and minimal change GN). Irrespective of subgroup analyses, the distribution of the most prevalent ethnicities was comparable in the no-DN and BP-DN groups as determined by means of *χ*^2^ test. Also, the frequency of (CTG)_5_ homozygosity did not differ between the two major ethnicities, German and Turkish.

It should be noted that in the group of BP-DN, DM duration was shorter (time from DM diagnosis: 14 (9–20) years versus 21.0 (15–29) years, BP-DN versus CIC-DN) with more severe hyperglycemia and albuminuria (HbA1c: 7.6 (6.7–8.8) % versus 7.3 (6.8–8.2) %, albuminuria: 2070 (337–3290) mg/l versus 644 (327–2110) mg/l, BP-DN versus CIC-DN).

### 3.2. Association of the CNDP1 (CTG)_5_ Homozygous Genotype with Diabetic Nephropathy

**Figure 2:**
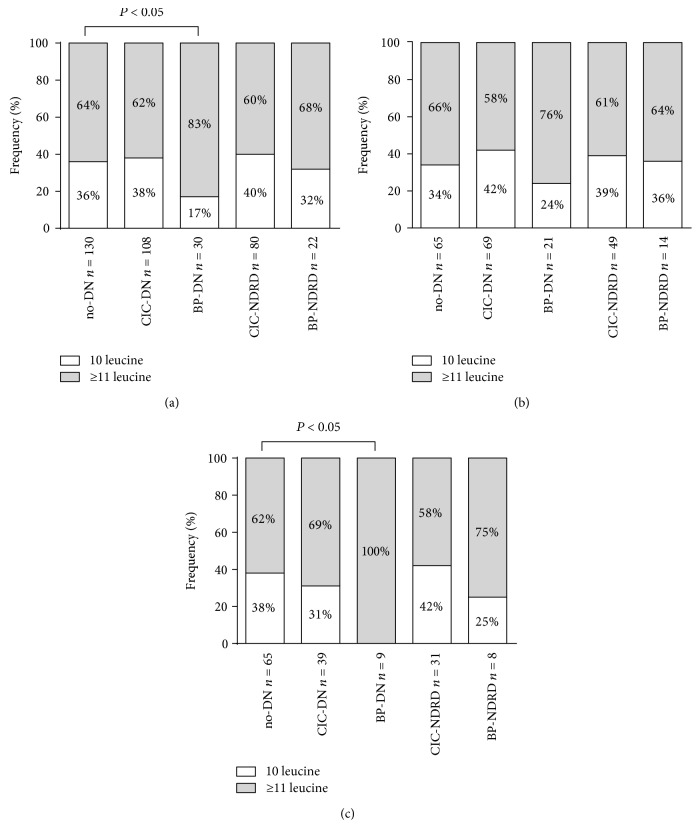
*CNDP1* (CTG)_*n*_ genotype distribution in T2DM patients. Genotype distribution is depicted as homozygosity for the (CTG)_5_ allele (10 leucine) versus all other genotypes (≥11 leucine). Planned comparisons were carried out between T2DM patients without DN and with either CIC- or BP-defined nephropathy. (a) No significant difference in genotype distribution was observed between T2DM patients with DN and without DN when applying CIC. The frequency of patients homozygous for the (CTG)_5_ allele decreased when BP-DN was considered. However, this difference did not hold after Bonferroni correction. ((b) and (c)) Gender stratification ((b) male patients, (c) female patients) showed no significant difference in the frequency of homozygosity for the *CNDP1* (CTG)_5_ allele between T2DM with and without DN when applying CIC. When DN was confirmed through biopsy, however, the frequency of *CNDP1* (CTG)_5_ homozygosity significantly decreased in female T2DM patients, which remained significant after Bonferroni adjustment.

If diagnosis of DN was based on CIC alone, the frequency of the homozygous (CTG)_5_ genotype did not significantly differ between the no-DN and CIC-DN groups (Figure 2(a), 36% versus 38%). The frequency of the protective genotype dropped to 17% when biopsy-proven DN was considered only (36% versus 17%; no-DN versus BP-DN, *P* < 0.05, NS after Bonferroni correction) (Figure 2(a)).

To confirm the previous findings of the sex-specific association between DN and the homozygous (CTG)_5_ genotype, patients were stratified according to gender. In male patients, neither CIC-DN nor BP-DN was associated with (CTG)_5_ homozygosity when compared to no-DN (no-DN: 34%, CIC-DN: 42%, BP-DN: 24%) (Figure 2(b)). Although the homozygous (CTG)_5_ genotype was less frequent in both the female CIC-DN and the female BP-DN groups as compared to the no-DN group, this difference was only significant for BP-DN (38% versus 0%, no-DN versus BP-DN, *P* < 0.05, significant after Bonferroni correction) (Figure 2(c)).

To confirm that (CTG)_5_ homozygosity is an independent, negative predictor for biopsy-proven diabetic nephropathy, multivariate logistic regression analysis was performed (Table 2). Seven diabetes-associated factors were selected as independent variables (age, BMI, diabetes duration, HbA1c, male sex, systolic blood pressure, and (CTG)_5_ homozygosity). All variables except BMI showed a *P* value below 0.25 in univariate analysis and were consequently included in the multivariate model. The Hosmer-Lemeshow test demonstrated an excellent goodness of fit (*χ*^2^ = 3949, *P* = 0.862) of the resulting multivariate model. The area under the receiver operating characteristic curve (ROC-AUC) further indicated adequate discrimination (AUC = 0.797).

The (CTG)_5_ homozygous genotype was significantly associated with biopsy-proven nephropathy in both univariate (OR = 0.353, *P* = 0.047) and multivariate analyses (OR = 0.307, *P* = 0.046) and, as such, identified as an independent protective factor. Interestingly, significance was reached despite the markedly lower number of cases (*n* = 30) generally believed to be necessary for obtaining sufficient power (i.e., *n* = 10/independent variable). In the multivariate model, systolic blood pressure (OR = 1.024, *P* = 0.045) was positively and age (OR = 0.931, *P* = 0.007) and diabetes duration (OR = 0.895, *P* = 0.025) were negatively associated with biopsy-proven nephropathy, respectively.

### 3.3. CNDP1 Genotype Distribution over Time on Hemodialysis and Diabetes Duration

The *CNDP1* genotype distribution (*CNDP1* (CTG)_5_ homozygous—versus all other *CNDP1* genotypes) was tested in 175 patients on hemodialysis, including 90 patients with DN according to CIC and/or BP-DN and 85 CIC-NDRD patients. Patients were stratified on the basis of hemodialysis duration, that is, time on dialysis: <30 months (*n* = 60), between 30 and 100 months (*n* = 76) and >100 months (*n* = 39). To assess the frequencies over time on hemodialysis and diabetes duration, the *χ*^2^ test for trend was carried out.

**Figure 3:**
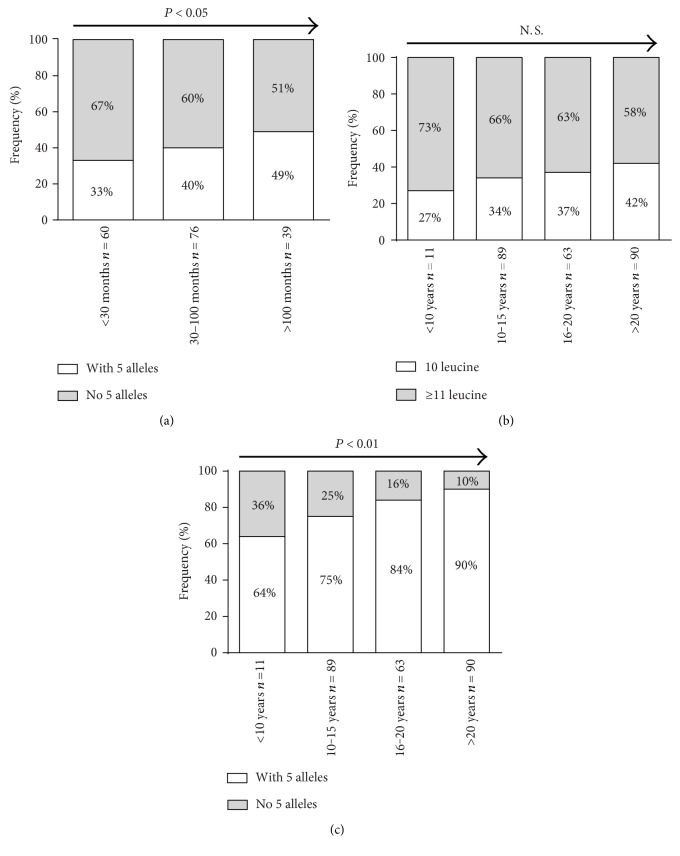
The *CNDP1* (CTG)_5_ genotype distribution is changed with time on dialysis and diabetes duration. To assess if the frequencies change over time, the *χ*^2^ test for trend (Cochran-Armitage test for trend) was carried out. (a) The frequency of the homozygous *CNDP1* (CTG)_5_ genotype (10 leucine) significantly increased with time on hemodialysis. ((b) and (c)) Although the observed frequency of the homozygous (b) *CNDP1* (CTG)_5_ genotype uniformly increased with diabetes duration, this trend did not reach statistical significance. Yet, if patients with a heterozygous (c) *CNDP1* (CTG)_5_ genotype (one 5 allele) were included as well, a significant trend towards high frequencies with increasing diabetes duration was found. N.S.: not significant.

The frequency of the (CTG)_5_ homozygous genotype significantly increased with time on hemodialysis (<30 months: 33%, 30–100 months: 40%, >100 months: 49%, *P* < 0.05) (Figure 3(a)), while gender distribution was approximately equal in the groups. The association of the (CTG)_5_ homozygous genotype with time on hemodialysis was not significant in the subgroup analyses after gender stratification (contingency tables not shown).

Although the frequency of the homozygous *CNDP1* (CTG)_5_ genotype uniformly increased with diabetes duration, this trend did not reach statistical significance (<10 years: 27%, 10–15 years: 34%, 16–20 years: 37%, >20 years: 42%, *P* = 0.17) (Figure 3(b)). However, if patients with a heterozygous *CNDP1* (CTG)_5_ genotype were included (i.e., patients with at least one (CTG)_5_ allele), there was a clear, significant trend towards high frequencies with increasing diabetes duration (<10 years: 64%, 10–15 years: 75%, 16–20 years: 87%, >20 years: 90%, *P* < 0.01) (Figure 3(c)).

### 3.4. CN-1 Activities in Hemodialysis Patients

**Figure 4:**
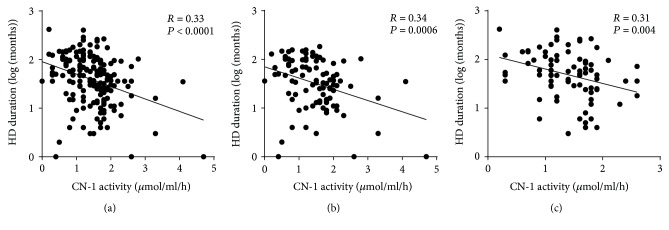
CN-1 activities decrease with time on dialysis. (a) Serum CN-1 activity was assessed in 175 hemodialysis patients and plotted against the log-transformed duration since hemodialysis was initiated. A significant correlation between serum CN-1 activity and log-transformed hemodialysis duration was found in all patients. ((b) and (c)) After stratification in T2DM (b) and other causes of renal failure (c), the correlation remained significant. HD: hemodialysis.

Because serum carnosinase (CN-1) activity correlates with *CNDP1* (CTG)_*n*_ genotypes, that is, CN-1 activity is in general lower in individuals with less CTG copies, we also cross-sectionally assessed if serum CN-1 activity changes with time on dialysis. In line with the increased frequency of the homozygous *CNDP1* (CTG)_5_ genotype in the groups of patients with a long history of hemodialysis, a significant negative correlation between serum CN-1 activity and log-transformed hemodialysis duration was found in all patients (*r* = −0.33; *P* < 0.0001, Figure 4(a)), T2DM patients only (*r* = −0.034, *P* = 0.0006, Figure 4(b)), and nondiabetic hemodialysis patients (*r* = −0,031, *P* = 0.004, Figure 4(c)).

**Figure 5:**
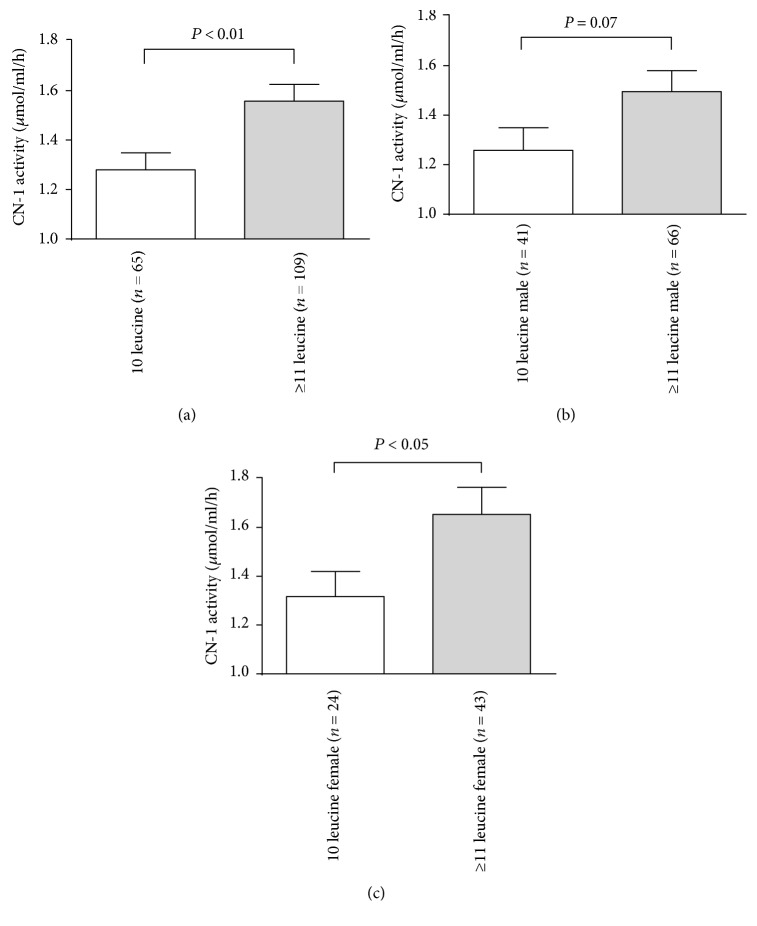
CN-1 activity correlates with *CNDP1* genotype in hemodialysis patients. (a) CN-1 activity in (CTG)_5_ homozygous hemodialysis patients is significantly lower than that in patients carrying other genotypes. ((b) and (c)) After gender stratification differences in CN-1 activity between the (CTG)_5_ homozygous, all other genotypes remained but only reached statistical significance in females (c).

To delineate if the *CNDP1* genotype is relevant for CN-1 activities in hemodialysis patients, patients on hemodialysis were stratified on the basis of homozygosity for the (CTG)_5_ allele. Out of 174 subjects, the 65 patients carrying the homozygous *CNDP1* (CTG)_5_ genotype showed a significantly lower serum CN-1 activity compared to patients with other genotypes (Figure 5(a), *P* < 0.01). This remained significant in female (Figure 5(c), *P* < 0.05), but not in male (Figure 5(b), *P* = 0.07) patients after gender stratification.

## 4. Discussion

This study examined whether the protection against DN afforded by the homozygous *CNDP1* (CTG)_5_ genotype is still observed when applying clinical inclusion criteria or biopsy findings only and if the prevalence of the protective genotype changes in situations of increased cardiovascular mortality. Our results demonstrate that the frequency of the homozygous *CNDP1* (CTG)_5_ genotype in the group of patients with biopsy-proven DN is significantly lower as compared to the groups of patients with no DN or with other biopsy-proven nephropathies. Our study also indicates that the frequency of homozygous *CNDP1* (CTG)_5_ genotype tends to be higher in patients with a longer duration of hemodialysis, particularly in female patients. An analogous increase was detected for patients carrying at least one (CTG)_5_ allele stratified for diabetes duration.

CIC for group allocations in DN studies bears the risk of wrongly assigning patients to the DN group as up to 20–50% of diabetic patients with albuminuria develop NDRD without concurrent DN [25, 26]. Although the presence of diabetic retinopathy (DR) is helpful for the prediction of DN [27] and thus improves the validity of group allocation, still DR may be absent in up to 50% of DN patients [28, 29]. Controversial studies reporting on genetic susceptibility loci for DN, including those for the *CNDP1* (CTG)_*n*_ polymorphism, might partly underlie this problem.

The use of large cohorts from different consortia and subsequent meta-analysis of data obtained from genome-wide association studies (GWAS) may partly overcome this problem as the proportion of wrongly allocated patients might be outnumbered by the large number of studied patients. The GWAS approach has been successfully utilized in newer studies confirming susceptibility loci for declining glomerular filtration rate (eGFR) or albuminuria [30–32]. The *CNDP1* locus, a postulated DN susceptibility locus found by positional cloning [5] and case control studies [10], has never been reported to be linked to DN in a GWAS approach, despite the fact that other genetic studies [11, 14, 33, 34] including a meta-analysis on 4546 DN, 7994 diabetes mellitus (DM), and 1826 healthy subjects [35] have confirmed an association between the *CNDP1* (CTG)_*n*_ polymorphism and DN in T2DM patients. Significance further increased in these studies if more stringent CIC for DN, for example, the presence of proliferative DR and a longer duration of T2DM, were considered [11].

In our study, the association between the protective homozygous *CNDP1* (CTG)_5_ genotype and DN was restricted to biopsy-proven DN and only significant in female patients using the *χ*^2^ test. Multivariate logistic regression subsequently identified (CTG)_5_ homozygosity as an independent protective factor for biopsy-proven DN with an odds ratio of approximately 0.3. The negative association of age and diabetes duration with biopsy-proven DN in this analysis may be explained by the fact that in older diabetic patients, a renal biopsy is often waived due to the lack of consequence.

The frequencies of the homozygous *CNDP1* (CTG)_5_ genotype in the no-DN and BP-NDRD groups were comparable, suggesting that this genotype does not afford protection against NDRD. Nonetheless, it would be prudent to be cautious with this assumption as other studies have suggested that this genotype also affords protection against other chronic kidney diseases (CKD), for example, glomerulonephritis but not tubulointerstitial nephritis [36]. In this light, the paradox between CIC-DN and BP-DN might be due to the fact that patients with NDRD were falsely included in the CIC-DN group, underscoring that clinical criteria do not provide a sufficient certainty for the diagnosis of DN in T2DM patients. It is important to note that patients in the BP-DN group had a shorter DM duration and displayed more severe hyperglycemia and albuminuria as compared to the CIC-DN group. Because of this relatively atypical DN course, these patients required a biopsy to clarify the actual underlying renal disease. Whether the change in *CNDP1* genotype distribution between CIC-DN and BP-DN underlies the severity of disease per se is unknown so far and cannot be excluded.

Our data are in agreement with a previous publication showing that the association of *CNDP1* and DN is most likely sex-specific [14]. Although also in males of the BP-DN group the frequency of the (CTG)_5_ homozygous genotype was lower as compared to the no-DN group, it was only significantly decreased in female BP-DN patients. The sex-specific protection of *CNDP1* is generally explained by higher serum CN-1 activities in females [14].

In keeping with the recently published prospective study that the *CNDP1* genotype may impart a cardiovascular mortality risk in female, but not in male T2DM patients [19], we investigated whether genotype distribution changes with time on dialysis or diabetes duration. Since both of the latter variables are associated with an increased (cardiovascular) mortality risk, it would be expected based on the above study that the frequency of the homozygous *CNDP1* (CTG)_5_ genotype would decrease rather than increase in patients with a long history of hemodialysis. By contrast, our data show that the frequency of the homozygous *CNDP1* (CTG)_5_ genotype was significantly increased in patients with a long history of hemodialysis. This difference remained in both males and females although it was not statistically significant, which is likely explained by the small sample size of the subgroup analysis. Similar findings were also observed with respect to a longstanding diabetes duration when all patients carrying at least one (CTG)_5_ allele were included. Although our findings suggest that in patients on hemodialysis and in diabetic patients the *CNDP1* (CTG)_5_ genotype may not impart an additional mortality risk, it should be underscored that the small group sizes and the cross-sectional design of this study impede drawing firm conclusions, in particular since a considerable number of hemodialysis patients were not diabetic. Nonetheless, these findings suggest that in patients on hemodialysis and in diabetes, the *CNDP1* (CTG)_5_ genotype may not impart an additional mortality risk.

Serum CN-1 concentrations and activities are in part determined by (CTG)_*n*_ polymorphism [10, 37]. Since this repeat is located in the hydrophobic part of the CN-1 signal peptide and is essential for the translocation of CN-1 protein during secretion, it is believed that the shorter (CTG)_5_ variant is less efficiently secreted [37]. In line with an increased frequency of the homozygous *CNDP1* (CTG)_5_ genotype in patients with a long history of hemodialysis, CN-1 activities were reduced. This reduction is not due to the loss of protein through hemofiltration since serum CN-1 concentrations even increase proportionally to the amount of ultrafiltrate [38].

As discussed above, we acknowledge the relatively small sample size as a major limitation of our study, accounting for a limited statistical power regarding major questions addressed. In addition, in contrast to a prospective study design, no systematic biopsy strategy with uniform indications and assigned nephropathologists could be implemented. Other studies, however, demonstrated that the histological classification of DN based on glomerulopathy shows a satisfying interobserver reproducibility [39]. We also acknowledge uncertainties regarding the procedure of patient allocation leading to limited group selectivity. This holds true especially for the diabetic control group without DN, which is based only on clinical criteria instead of a histological diagnosis. As 80% of these patients were on ACE inhibitor or AT_1_-blocking drugs, albuminuria alone was not a reliable parameter. Despite the extension of the criteria by diabetes duration of >15 years and exclusion of patients which manifest retinopathy, accidental assignment of patients with DN to this group is not improbable.

In our eyes, the fact that this study still resulted in significant results in the light of these conceptual drawbacks supports a particular strong association of our findings. Our study supports the hypothesis that protection against DN is indeed afforded by the *CNDP1* (CTG)_5_ genotype and that this association mainly applies to female T2DM patients. The restriction of this finding to the BP-DN group may be attributed to false allocation of patients with other proteinuric diseases to the CIC-DN group. In fact, 20–50% of diabetic patients with proteinuria display NDRD without concurrent DN [25, 26]. Our investigation also suggests that (CTG)_5_ homozygous hemodialysis patients and patients with diabetes carrying at least one (CTG)_5_ allele might have a survival benefit as compared to other genotypes. These findings warrant further conformational studies, ideally with a prospective longitudinal design.

## Figures and Tables

**Table 1:** Demographic and clinical data of all patients.

	No-DN	CIC-DN	BP-DN	CIC-NDRD	BP-NDRD
*N*	130	108	30	80	22
*Demographic characteristics*					
Male sex—*n* (%)	65 (50)	69 (64)	21 (70)	49 (61)	14 (64)
Age—year	71 (63–75)	71 (62–76)	61 (57–69)	61 (48–74)	61 (55–78)
*Clinical characteristics*					
Body mass index—kg/m^2^	31.1 (28–35)	29.8 (27–35)	29.9 (25–35)	24.5 (21–27)	27 (24–32)
Hypertension					
Number of AHM	3 (2–3)	3 (2–4)	3 (2–5)	3 (1.5–4)	2 (2–4)
Blood pressure—mmHg					
Systolic	129 (120–140)	135 (120–156)	140 (130–150)	135 (120–145)	140 (128–153)
Diastolic	70 (66–80)	70 (60–80)	75 (70–80)	70 (60–80)	70 (64–80)
*Diabetes mellitus*					
Time from diagnosis—year	16 (13–20)	21 (15–29)	14 (9–20)	—	—
HbA1c—%	7.0 (6.4–8.1)	7.3 (6.8–8.2)	7.6 (6.7–8.8)	5.6 (5.4–5.7)	5.6 (5.1–6.2)
*Kidney function*					
Creatinine—mg/dl	0.9 (0.8–1.1)	6.3 (3.7–8.8)	5.7 (3.0–7.3)	9.7 (7.5–11.5)	3.4 (1.7–5.0)
eGFR—ml/min	73 (61–87)	9 (6–16)	10 (6–20)	5 (4–8)	15 (8–37)
Hemodialysis—*n* (%)	0 (0)	83 (75)	18 (60)	85 (100)	8 (36)
HD duration—months^∗^	0 (0–0)	26 (1–69)	3 (0–30)	56 (28–100)	0 (0–0.3)
Albuminuria—mg/l	9 (5–16)	644 (327–2110)	2070 (337–3290)	470 (261–1587)	556 (189–1308)
*Retinopathy* (*DR*)*—n* (*%*)					
No DR	107 (82)	1 (0)	6 (20)	—	—
NPDR	23 (18)	68 (63)	11 (37)	—	—
Proliferative DR	—	17 (16)	4 (13)	—	—
Maculopathy	—	8 (7)	3 (10)	—	—
Laser therapy	—	13 (12)	3 (10)	—	—
Polyneuropathy—*n* (%)	54 (42)	56 (52)	17 (57)	—	—
*History*—*n* (*%*)					
Coronary heart disease	42 (32)	74 (69)	11 (37)	24 (30)	6 (22)
Cardiovascular event	20 (15)	42 (39)	7 (23)	14 (18)	5 (23)
Arterial occlusive disease	24 (18)	56 (52)	11 (37)	17 (21)	3 (14)
Stroke	19 (15)	28 (26)	6 (20)	8 (10)	2 (9)
Statin	86 (66)	79 (73)	19 (63)	28 (35)	10 (45)
Homozygous CTG_5_—*n* (%)	47 (36)	41 (38)	5 (17)	32 (40)	7 (32)

^∗^Patients on hemodialysis only. Categorical data are represented as numbers (%) and continuous data as median with corresponding 25th and 75th percentiles (IQR). AHM: antihypertensive medication; eGFR: estimated glomerular filtration rate; HD: hemodialysis; DR: diabetic retinopathy; NPDR: nonproliferative diabetic retinopathy.

**Table 2:** Summary of logistic regression analysis of variables predicting biopsy-proven diabetic nephropathy (male and female, *n* = 160).

	Univariate analysis			Multivariate analysis		
	OR	95% CI	*P* value	OR	95% CI	*P* value
Age (years)	0.920	0.881–0.961	<0.001	0.931	0.883–0.980	0.007
BMI (kg/m^2^)	0.980	0.922–1.041	0.513	—	—	—
Diabetes duration (years)	0.886	0.813–0.966	0.006	0.895	0.812–0.986	0.025
HbA1c (%)	1.308	1.020–1.678	0.034	1.169	0.844–1.619	0.348
Male sex	2.333	0.994–5.477	0.052	1.957	0.748–5.124	0.171
SBP (mmHg)	1.021	1.000–1.042	0.052	1.024	1.001–1.047	0.045
CTG_5_ homozygosity	0.353	0.127–0.984	0.047	0.307	0.096–0.980	0.046

Area under the ROC curve (AUC) = 0.797, *P* = 0.862 for Hosmer-Lemeshow test. BMI: body mass index; SBP: systolic blood pressure.
